# Analysis of early diagnosis methods for asymmetric dementia in brain MR images based on genetic medical technology

**DOI:** 10.1515/biol-2022-0690

**Published:** 2023-09-02

**Authors:** Xiao Zhang, Ning Tang, Yanlin Yin, Jian Zhou, Rui Jiang, Jinping Sheng, Jing Zhu

**Affiliations:** Department of Radiology, The General Hospital of Western Theater Command, Chengdu 610083, Sichuan, China; Department of Radiology, Joint Security Forces 945 Hospital, Yaan 625000, Sichuan, China

**Keywords:** dementia diagnosis, magnetic resonance, Alzheimer’s disease, support vector machine, early diagnosis, feature extraction

## Abstract

Alzheimer’s disease (AD) is a relatively common senile neurodegenerative disease and the main manifestation of senile dementia. In the pathological changes of AD, the asymmetry of the brain also changes. Therefore, finding an early diagnosis method of AD based on asymmetry is the key to the treatment of Alzheimer’s. Magnetic resonance (MR) imaging can quantitatively reflect the structural and functional changes of various tissues in the brain. It has the advantages of non-invasive, high spatial resolution, and non-radiation, and has been widely used in the early diagnosis of AD. In this work, asymmetric images were extracted from multiple brain MR images, and different morphological and texture features were extracted. By establishing a feature selection classification integration model, image features in the image were deeply fused to obtain higher and more stable recognition results than before. By filtering image samples, the corresponding sample feature matrix was obtained. Support vector machine was used for classification, and its classification accuracy had improved significantly compared with that before selection. In the experimental data of normal control group and AD group, the accuracy, sensitivity, and specificity of the feature selection algorithm were 93.34, 90.69, and 95.87%, respectively. In the normal control group and the mild cognitive impairment group, the accuracy, sensitivity, and specificity of the feature selection algorithm in this work were 85.31, 79.68, and 88.54%, respectively. On the whole, the classification accuracy of the feature selection algorithm in this work was much higher than that of other items. In addition, from the classification ability and distribution of asymmetric features, it can be seen that this asymmetric feature had a more significant consistent diagnostic role in clinical practice.

## 1 Introduction

With the increasing aging of the global population, the incidence of Alzheimer’s disease (AD) has increased significantly, which brings a heavy economic burden to patients, caregiver families, and society. Its main symptoms include progressive memory disorder, visuospatial skill disorder, executive dysfunction, and personality and behavior changes. AD is a relatively common neurodegenerative disease and a relatively common neurogenic dementia with great harm. Therefore, effective prevention and treatment of AD must be non-invasive in the early stage. Relevant research shows that the image shape and texture features of AD have some relationship with its early lesions, but the relationship between these features is very complex, so it is necessary to select fusion. In this study, many morphological and texture features are extracted from Magnetic resonance (MR) images and divided into different features.

With the continuous development of society, the research on dementia diagnosis is gradually increasing. Arvanitakis et al. found that AD is a cause of dementia in the United States, affecting 5.8 million people. The diagnosis of dementia requires a historical assessment of cognitive decline and impairment in daily activities and confirmation from close friends or family members [1]. Astell et al. summarized the key areas of dementia technology development and determined the future direction and impact. Members of the American Alzheimer’s Association in the field of technical expertise participated in the annual pre-session meeting and summarized the existing knowledge of the current and future development of dementia technology [2]. The research report of Kramarow and Tejada-Vera provided data on dementia mortality. From 2000 to 2017, the data of dementia as a potential cause of death were shown by specific characteristics such as age, sex, race, Hispanic descent, and living status [3]. Wittenberg et al. measured the average per capita and total annual costs of AD in the UK in 2015, in order to better support the patients with dementia and their caregivers, as well as to provide fair and effective funding for social care services, which is crucial to address the current and future challenges of dementia [4]. Livingston et al. added three risk factors for dementia with new and convincing evidence. These factors include excessive drinking, traumatic brain injury, and air pollution [5]. Although these studies have promoted the diagnosis of dementia to some extent, they have not been combined with the actual situation.

At the same time, MR imaging has gradually attracted widespread attention from the academic community. Ravindranath and Sundarakumar reviewed the literature on the epidemiology and risk factors of dementia in the Indian population, and discussed the work to be carried out in the future, so as to implement public health interventions and reduce the burden of dementia, which provided a reference for the use of MR imaging [6]. Based on the consideration of the existing literature, Baird and Thompson outlined a new theoretical framework to explain the relationship between music and self in patients with dementia, which promoted the use of MR imaging [7]. Van Der Flier et al. provided an overview of the results based on the Amsterdam dementia cohort, and described the main results of these studies: early diagnosis, heterogeneity, and vascular factors, which affected the application effect of MR imaging [8]. Peters et al. systematically reviewed the evidence basis of the relationship between air pollution and cognitive decline and dementia, which provided ideas for the improvement of MR imaging [9]. Although these research methods are innovative, a large number of experimental data are needed to prove the reliability of the methods.

In this study, the methods of brain MR image recognition were analyzed first, including brain MR image preprocessing, MR image feature extraction, MR image recognition classification, and brain MR asymmetric feature extraction. Second, the conditions of the experiment in this study were explained. On this basis, the experimental research was carried out. Finally, the classification and recognition results, and the related experiments of the comparison of the classification and recognition results before and after feature selection were analyzed.

## 2 Brain MR image recognition method

### 2.1 Brain MR image preprocessing

MR imaging is an imaging examination technique. Since the 1980s, MR imaging technology has been greatly developed in more than 20 years. It has made considerable progress in software and hardware, and has been widely used in clinical practice, providing strong support for many important clinical problems. MR imaging is safe, radiation-free, and can be imaged at any angle. Its image can show good tissue contrast. In addition to morphological analysis of flow rate, biochemical changes, and metabolic function, it can also be detected.

**Figure 1:**
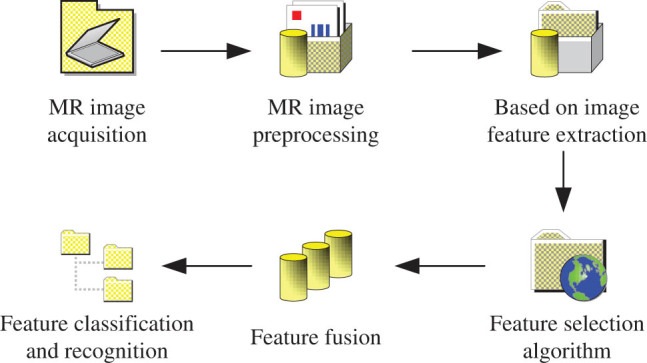
Flow chart of disease auxiliary diagnosis.

MR image feature fusion technology is to extract corresponding tissue features and perform fusion training according to the changes in the relevant tissues in the brain of AD patients, the characteristics of MR images, and the shape and size of tumors. Finally, three different types of brain MR images of AD group, mild cognitive impairments (MCI) group, and normal control group are recognized [10,11]. This study has designed a flow chart of disease aided diagnosis to achieve feature extraction, as shown in Figure 1.

For the obtained brain MR images, according to the flow chart in Figure 1, the image features in the tested tissues should be collected first. After that, preprocessing of MR images is performed and then features are selected based on the volume texture and other features of the images. The extracted features of multiple categories are fused and filtered to obtain the subset with the largest feature. The classifier is used for training, and the final classification result is obtained and analyzed.

The purpose of image preprocessing is to improve the quality of the whole image and eliminate the interference and noise generated by medical instruments when acquiring images, thus causing different spatial positions. This process is mainly divided into image registration, noise, and segmentation.

**Figure 2:**
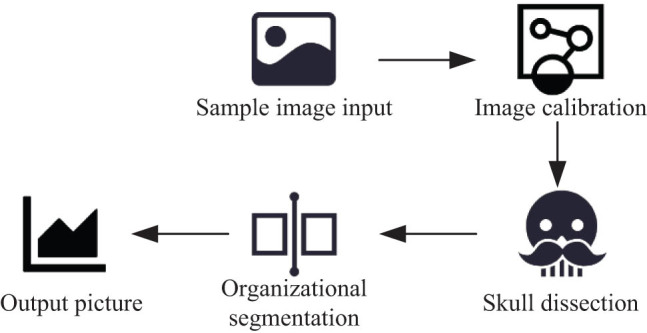
MR image preprocessing.

Due to different acquisition environments, different images have certain differences in different directions and positions, which would affect the accuracy of subsequent feature extraction and classification recognition. Therefore, the 3D images must first be rigidly aligned, so as to eliminate the spatial distribution of individual brain MR images and their topological relationships. The brain tissues collected in this study include scalp, gray matter, skull, white matter, glia, subcutaneous fat, cerebrospinal fluid, muscle, connective tissue, etc. [12]. However, due to the existing relevant tissues in the known brain tissues, other unrelated tissues such as skull and cerebellum must be removed. After the completion of the alignment, the cranial dissection needs to be performed. In the diagnosis of AD, this study proposes an asymmetric model based on the left and right brain, and divides it into two parts to classify the two brains, respectively, so as to improve the accuracy of recognition [13,14]. The image preprocessing process carried out in this study is shown in Figure 2.

### 2.2 MR image feature extraction

**Figure 3:**
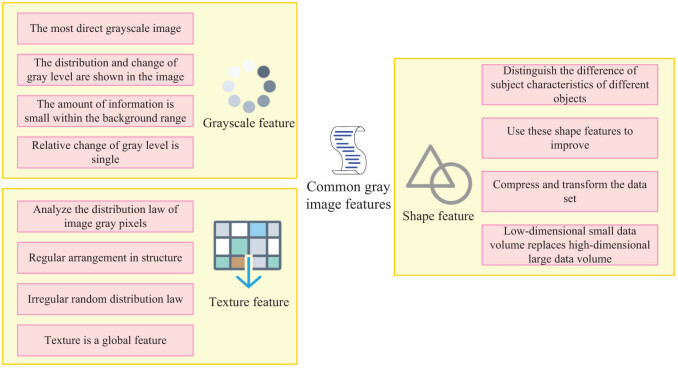
Common grayscale image features.

There are many kinds of feature classification of MR images, which can be divided into qualitative and quantitative categories according to their characteristics. Quantized features refer to the characteristics of an image that can be quantified by a function or a mathematical formula, such as variance gray and degree average. The qualitative characteristics cannot be quantified. They need to be expressed in pictures and words, such as semantics. MR images can be divided into local images and overall images by region. The global feature can reflect the local characteristics of the whole object, and the local feature is to divide the whole image into several small pieces according to certain rules. It divides the area of concern into different areas, and then separates the characteristics of these areas separately. As most medical images are gray images, this article focuses on the common gray image features as shown in Figure 3.

Grayscale characteristics: Feature grayscale is the most intuitive grayscale image. Its grayscale distribution and change are reflected in the image. Therefore, its information is mainly reflected through the change distribution of grayscale. Based on the pixel gray level as the feature, this method reflects the distribution of each gray level in the image by constructing a gray level histogram, and gives the corresponding mean, variance, energy, entropy, and other features. In this study, the gray level and distribution of images are classified, and they are collectively called gray level features, which have been widely used in image filtering, edge detection, and other aspects.

Texture property: Texture property is a property used to analyze the distribution rules of gray points in an image. Their distribution can be regular or irregular. If the image information is understood as a kind of overall visual sense with a certain uniform distribution, rather than simply viewing it as several individual objects, its overall sense is texture. In terms of structure and gray level, the change in texture has great regularity. Therefore, it is global, and generally presents the characteristics of density, gray level, uniformity, regularity, directionality, frequency, thickness, linearity, phase, and attribute. These regular features and changes can be quantitatively expressed as multi-scale features, frequency domain transformation features, correlation features, and gray statistical features.

Texture primitives are the basis of texture. According to the distribution and shape of textures, different textures can be constructed according to certain statistical rules. According to certain rules, different textures can be combined [15]. Different materials can be combined according to specific rules. According to different attributes, different materials can be divided into random texture and determined texture. Texture is a statistical property. A variety of parameters can be used to represent the texture’s roughness, density, strength, direction, and so on.

Shape feature: People distinguish different objects according to shape feature. Shape feature is an important image feature, often including ellipse, rectangle, curve, and other shape features, which is widely used in computer detection and recognition through shape features, such as recognition accuracy. According to different forms of expression, morphological features can be divided into two types: one is region-based, and the other is edge-based. The shape features are divided according to all the areas included, comprising the aspect ratio, invariance, area, etc., of the shape. According to the shape features of the boundary, these features include: Gaussian parametric curve, Fourier descriptor, polygon approximation line, line segment description, etc.

The so-called feature extraction is to compress and transform the data in the dataset according to specific rules, and convert it into data with lower dimensions. According to different extraction rules, features can be divided into two types. One is to use mathematical analysis to predetermine a criterion for judging a feature and to process any feature on this basis, so that the maximum value can be reached. The second method is to characterize the experience and intuition of the target object, and find the characteristics of the target object through experiments.

At present, the statistical feature extraction method is the mainstream method of image feature extraction, including co-occurrence matrix, correlation function, gray distribution statistics, gray histogram statistics, etc. The features selected by gray histogram and gray distribution statistics are very representative. However, because the spatial grayscale relationship in the image cannot be fully reflected, and the grayscale distribution of the image corresponding to the last set of the same histogram results would also have great differences, this method cannot completely show the characteristics and spatial distribution of the image. The different order geometric moments of the image can reflect the various gray space properties of the image, and the rotation translation has no effect on these features, while the corresponding transformation in the moment space can more easily reflect the coordinate transformation of the image. Therefore, using the characteristics of moment method has good application effect. The correlation function method and the co-occurrence matrix method are realized through the comprehensive analysis of the spatial relationship of pixel gray level, which can well reflect the relevant spatial properties of gray level, so they are also widely used in texture images.

### 2.3 MR image recognition and classification

MR image recognition technology is a supervised method to classify MR images. This method usually trains different types of images and constructs a conceptual model that can describe these images, such as rules, formulas, etc., which are used to classify and label unlabeled images.

Common classification methods include neural network, decision tree classification, Support vector machine (SVM), etc. These methods have their advantages and disadvantages. The advantage of decision tree classification is that it is easy to convert into classification rules, and the operation speed is fast. However, its scalability is not strong, and the depth operation must be carried out first. Therefore, the calculation amount of this algorithm is limited and cannot effectively process massive data. This method has the advantages of nonlinear fitting, good adaptability, effective mapping of complex objects with nonlinear relations, easy computer implementation, and good self-learning, nonlinear mapping, and memory ability, so its application prospect is broad. The principle of SVM is to minimize the structural risk. It has strong generalization ability and approximation ability, good nonlinear mapping ability, global optimality of the algorithm, support for small sample statistics, and other advantages.

SVM is of great significance in machine learning and statistical research. The core idea of SVM was put forward in 1992. SVM was put forward based on the study of the best classification surface in linear separable problems.

The central idea of SVM is to maximize the classification interval, so as to control the scalability. According to the principle of statistical learning, the Vapnik–Chervonenkis dimension of the indicator function set \[f(x,w,q)=\mathrm{sgn}({w}^{T}x+q)]\] (in which sgn is the sign function) of the regular hyperplane combination conforming to the condition \[\parallel w\parallel \le S]\] is set in the hypersphere region of *R* in the *M*-dimensional space, which satisfies formula (1) as follows:

(1) \[h\le \hspace{.25em}\min ({[}{R}^{2},{S}^{2}],M)+1.]\]

The optimal classification function is expressed as formula (2).

(2) \[f(x)=\mathrm{sgn}\left\{\phantom{\rule[-1.05em]{}{0ex}}\mathop{\sum }\limits_{o=1}^{m}{\beta }_{1}^{\ast }{y}_{o}({x}_{o}^{T}x)+{q}^{\ast }\right\}.]\]

The classification threshold is expressed by \[{q}^{\ast }]\] .

In SVM, different methods generate corresponding inner product kernel functions, which usually use three kinds of kernel functions. The first is polynomial kernel function, and its result is polynomial classification of degree *u*, as shown in formula (3). The second is the radial basis function, which has a support vector at the center of each basis function and determines its output weight through this algorithm. The third is to use the inner product function of sigmoid. What SVM needs to do is to implement a multi-layer perceptron with hidden layers, which can automatically determine the number of hidden layers through algorithms.

(3) \[K(x,{x}_{o})={({x}^{T}{x}_{o}+1)}^{u},]\]

(4) \[K(x,{x}_{o})=\exp \left\{-\frac{{| x-{x}_{o}| }^{2}}{{u}^{2}}\right\},]\]

(5) \[K(x,{x}_{o})=\hspace{.25em}\tanh (C({x}^{T}{x}_{o})+C).]\]

According to the relevant literature, the radial basis kernel function has better performance than the other two methods, and the calculation amount is also less.

### 2.4 Brain MR asymmetric feature extraction

The results show that the anatomical structure of AD patients has significant changes [16,17]. In addition, in the early stage of AD, the asymmetry of the brain is also gradually changing [18,19]. On this basis, the volume characteristics and texture characteristics of four different anatomical parts of the brain are analyzed. The eight volume features are left brain gray matter volume, right brain gray matter volume, left brain white matter volume, right brain white matter volume, left brain cerebrospinal fluid volume, right brain cerebrospinal fluid volume, left brain hippocampus volume, and right brain hippocampus volume. Texture features include gray level co-occurrence matrix and run-length matrix. Among them, there are five features of gray level co-occurrence matrix.

Energy:

(6) \[{F}_{\text{Energy}}=\mathop{\sum }\limits_{{o}_{g}=0}^{Z-1}\mathop{\sum }\limits_{{k}_{g}=0}^{Z-1}{{[}{P}_{g}({o}_{g},{k}_{g},d,\varphi )]}^{2}.]\]

Contrast:

(7) \[{F}_{\text{constrast}}=\mathop{\sum }\limits_{{o}_{g}=0}^{Z-1}\mathop{\sum }\limits_{{k}_{g}=0}^{Z-1}{({o}_{g}-{k}_{g})}^{2}{P}_{g}({o}_{g},{k}_{g},d,\varphi ).]\]

Contrast reflects the clarity of image texture. The higher the contrast, the clearer the texture. The function reflects the distance between the largest element in the matrix and the main diagonal. High-value points in coarse texture are mainly concentrated near the main diagonal, and \[{({o}_{g}-{k}_{g})}^{2}]\] in this range is small, so the contrast is small. The fine texture corresponds to a greater contrast.

Inverse difference moment:

(8) \[{F}_{\text{IDM}}=\mathop{\sum }\limits_{{o}_{g}=0}^{Z-1}\mathop{\sum }\limits_{{k}_{g}=0}^{Z-1}\frac{{P}_{g}({o}_{g},{k}_{g},d,\varphi )}{1+{({o}_{g}-{k}_{g})}^{2}}.]\]

The inverse moment is an index reflecting the local uniformity of the image. The larger its value, the higher the local uniformity of the image.

Entropy:

(9) \[{F}_{\text{entropy}}=-\mathop{\sum }\limits_{{o}_{g}=0}^{Z-1}{P}_{g}({o}_{g},{k}_{g},d,\varphi )\log \hspace{.25em}{P}_{g}({o}_{g},{k}_{g},d,\varphi ).]\]

Entropy can be used to describe the complexity of texture. In the case of no texture, the matrix is approximately 0, and the entropy is approximately 0. When the image is filled with fine texture, all the elements in the co-occurrence matrix are equal and the entropy is the largest. In the case of rough texture, its \[{P}_{g}({o}_{g},{k}_{g},d,\varphi )]\] value changes greatly and its entropy is relatively low.

Correlation:

(10) \[{F}_{\text{correlation}}=\frac{1}{{\gamma }_{x}{\gamma }_{y}}\mathop{\sum }\limits_{{o}_{g}=0}^{Z-1}\mathop{\sum }\limits_{{k}_{g}=0}^{Z-1}{P}_{g}({o}_{g},{k}_{g},d,\varphi ){o}_{g}{k}_{g}-{\eta }_{x}{\eta }_{y}.]\]

\[{\eta }_{x}]\] and \[{\eta }_{y}]\] are gray level average and smooth average, respectively. Correlation description refers to the similarity of each element in the row or column direction in the gray level co-occurrence matrix. It reflects the extension of a certain gray level in a certain direction. The longer the extension is, the higher the correlation degree is, and vice versa. It is a measure of gray correlation degree.

The difference characteristics can reflect the difference of anatomical characteristics between brains. The calculation results of the difference characteristics are as follows:

(11) \[{\text{Fea}}_{o\_D}={\text{Fea}}_{o\_\text{left}}-{\text{Fea}}_{o\_\text{right}},]\]

(12) \[{\text{Fea}}_{o\_R}={\text{Fea}}_{o\_\text{left}}/{\text{Fea}}_{o\_\text{right}}.]\]

Among them, \[{\text{Fea}}_{o\_D}]\] and \[{\text{Fea}}_{o\_R}]\] represent the characteristic difference of the *o*-th characteristic and the characteristic ratio of the *o*-th characteristic, respectively; \[{\text{Fea}}_{o\_\text{left}}]\] represents the left feature of the *o*-th feature; \[{\text{Fea}}_{o\_\text{right}}]\] represents the right feature of the *o*-th feature.

## 3 Recognition experiment and evaluation of AD based on SVM

### 3.1 Experimental conditions

This method was verified by several experiments. First, the experimental conditions were given, and the main parameters of the method were given. Second, the classification accuracy of this method was obtained by analyzing various feature sets through multiple groups of experiments, which proved that the screening of asymmetric left brain is the most effective. Finally, the correctness of this method was proved by several experimental results. By studying the distribution of asymmetry and the monotonicity of individuals, the correlation between them and AD was discussed, which provided a reference for clinicians and researchers.

The data from people’s hospital were used in this experiment, and the data were true and reliable, which has been recognized by medical institutions.

Brief description of data information: These data included MR structural images with a total of 300 samplings. The acquisition equipment for the images was a 1.5T Signa scanner, and the acquisition was made before 2023. The acquisition method was the destructive gradient echo acquisition method, with an echo time of 10 ms, a repetition time of 30 ms, a reverse angle of 40°, the number of repeated excitations of one pulse, and a layer thickness of 1.5 mm.

The object of image acquisition was determined by experts from the Department of Neurology and Imaging of the People’s Hospital. The collected subjects had no other neurological diseases and had similar educational level. The number of AD samples was relatively small, and some of them were incomplete. Therefore, 50 complete AD samples were selected, while 50 were selected for each of the other two groups. There were a total of 150 samples, including image samples of AD, MCI, and normal control group. In tissue segmentation, the image was divided into gray matter, white matter, and cerebrospinal fluid. Hippocampal atrophy is a major feature of AD. Therefore, only the hippocampus was isolated from gray matter. Finally, the left and right sides of four different anatomical features were extracted.

### 3.2 Experimental evaluation

#### 3.2.1 Classification identification results

This study evaluated the asymmetric features and classifier performance selected by the algorithm in this study through accuracy (correctly classified samples divided by the total number of samples), sensitivity (correctly classified MCI [or AD] samples divided by the total number of MCI [or AD] samples), and specificity (correctly classified normal control group samples divided by the total number of normal control group samples). It was divided into two situations: the classification of normal control group and AD, and the classification of normal control group and MCI.

Whole brain feature: It is to synthesize the features of the left and right parts of the brain, and then screen the features to get the best feature subset for classification and recognition. Left brain feature: It is to synthesize the anatomical features of the left half, and then conduct feature screening to obtain the optimal feature subset for classification and recognition. Right brain feature: It is to integrate all the features of the right side of anatomy, and then conduct feature screening to obtain the optimal feature subset for classification and recognition.

**Figure 4:**
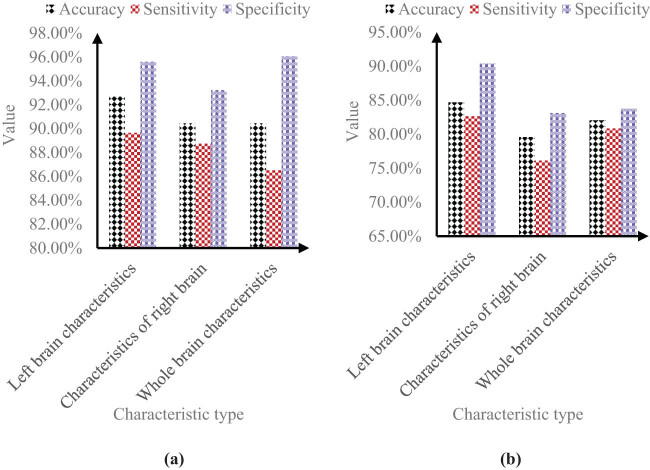
Classification recognition effect. (a) Normal control group and AD group and (b) normal control group and MCI group. It can be seen from Figure 4(a) that in the data of normal control group and AD group, the accuracy, sensitivity, and specificity of left brain features were 92.68, 89.65, and 95.60%, respectively; the accuracy, sensitivity, and specificity of right brain features were 90.45, 88.74, and 93.22%, respectively; the accuracy, sensitivity, and specificity of whole-brain features were 90.45, 86.51, and 96.06%, respectively.

Figure 4 shows the relevant data of classification recognition results.

It can be seen from Figure 4(b) that in the data of normal control group and MCI group, the accuracy, sensitivity and specificity of left brain features were 84.68, 82.64, and 90.38%, respectively; the accuracy, sensitivity, and specificity of right brain features were 79.57, 76.12, and 83.11%, respectively; the accuracy, sensitivity, and specificity of whole-brain features were 82.05, 80.85, and 83.74%, respectively.

It can be seen from the data that the classification of left brain features was more accurate than that of right brain features. This showed that the changes in the left brain of patients were more significant than those in the right brain. The classification of whole brain features for normal control group and AD, as well as the classification of normal control group and MCI was also lower than the classification of left brain features.

Feature difference: The absolute value of the difference is calculated from the left half value and the right half value according to the same feature, and is named as the feature difference. Ratio feature: It is to calculate the ratio of left and right half values based on the same feature, and this ratio is used as a new feature, known as the ratio feature.

Table 1 shows the relevant data of difference characteristics and ratio characteristics.

**Table 1:** Difference characteristics and ratio characteristics

Type	Normal control group and AD group		Normal control group and MCI group	
	Difference characteristic (%)	Ratio characteristic (%)	Difference characteristic (%)	Ratio characteristic (%)
Accuracy	86.12	85.83	77.63	77.68
Sensitivity	82.57	85.98	73.82	76.97
Specificity	89.75	84.34	82.86	79.70

It can be seen from Table 1 that in the data of the normal control group and the AD group, the accuracy, sensitivity, and specificity of the difference characteristics were 86.12, 82.57, and 89.75%, respectively, while the accuracy, sensitivity, and specificity of the ratio feature were 85.83, 85.98, and 84.34%, respectively. In the data of normal control group and MCI group, the accuracy, sensitivity, and specificity of the difference feature were 77.63, 73.82, and 82.86%, respectively, while the accuracy, sensitivity, and specificity of the ratio feature were 77.68, 76.97, and 79.70%, respectively.

#### 3.2.2 Comparison of classification and recognition results before and after feature selection

**Figure 5:**
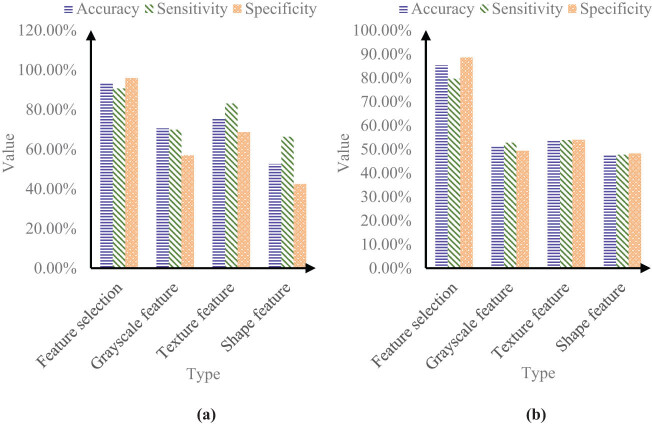
Classification and recognition performance before and after feature selection. (a) Data of normal control group and AD group and (b) data of normal control group and MCI group.

Figure 5 shows the relevant data of classification and recognition performance before and after feature selection.

According to Figure 5(a), in the data of normal control group and AD group, the accuracy, sensitivity, and specificity of the feature selection algorithm in this study were 93.34, 90.69, and 95.87%, respectively; the accuracy, sensitivity, and specificity of gray features were 70.52, 69.82, and 56.92%, respectively; the accuracy, sensitivity, and specificity of texture features were 75.68, 83.08, and 68.64%, respectively; and the accuracy, sensitivity, and specificity of shape features were 52.54, 66.27, and 42.48%, respectively.

According to Figure 5(b), in the normal control group and MCI group, the accuracy, sensitivity, and specificity of the feature selection algorithm in this study were 85.31, 79.68, and 88.54%, respectively; the accuracy, sensitivity, and specificity of gray features were 51.30, 52.77, and 49.32%, respectively; the accuracy, sensitivity, and specificity of texture features were 53.72, 53.86, and 53.93%, respectively; and the accuracy, sensitivity, and specificity of shape features were 47.68, 47.74, and 48.24%, respectively.

It can be seen from the data that the classification accuracy of the feature selection algorithm in this study was much higher than that of other items. In short, this method improved the accuracy of AD classification based on MR images.

### 3.3 Classification ability and distribution of asymmetric features

**Figure 6:**
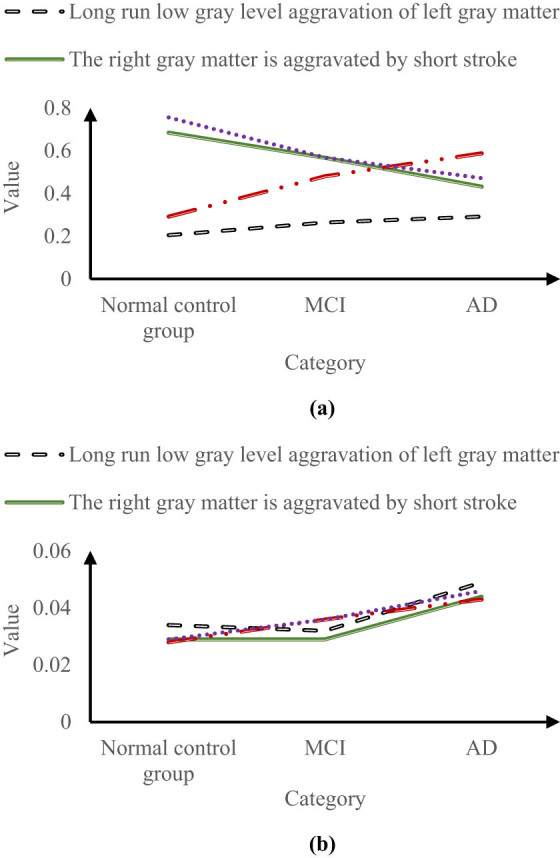
Partial eigenvalues in the optimal feature subset. (a) Mean value and (b) standard deviation.

The aim of this experiment is to test the recognition effect of each feature on three types after feature selection, so as to test the effectiveness of this method. On this basis, the characteristic values of three types of samples (normal control group, MCI, and AD) with four characteristics were selected for display. The results are shown in Figure 6.

According to Figure 6(a), in the normal control group, the mean values of long-run low-gray scale aggravation of left gray matter, short-run aggravation of right gray matter, low-gray scale aggravation of left gray matter, and short-run aggravation of left gray matter were 0.205, 0.685, 0.292, and 0.756, respectively; in the MCI group, the mean values of the four items were 0.264, 0.567, 0.481, and 0.568, respectively; in the AD group, the mean values of the four items were 0.292, 0.432, 0.588, and 0.472, respectively.

According to Figure 6(b), in the normal control group, the standard deviation of long-run low-gray scale aggravation of left gray matter, short-run aggravation of right gray matter, low-gray scale aggravation of left gray matter, and short-run aggravation of left gray matter were 0.034, 0.029, 0.028, and 0.029, respectively; in the MCI group, the standard deviations of the four items were 0.032, 0.029, 0.036, and 0.036, respectively; in the AD group, the standard deviations of the four items were 0.049, 0.044, 0.043, and 0.046, respectively.

It can be seen from Figure 6 that the characteristic mean values of long-run low-gray level aggravation of left gray matter and low-gray level aggravation of left gray matter monotonically increased with the change in category from normal control group to MCI and then to AD; the characteristic mean values of short-run aggravation of right gray matter and left gray matter monotonically decreased with the change in category from normal control group to MCI and then to AD.

**Figure 7:**
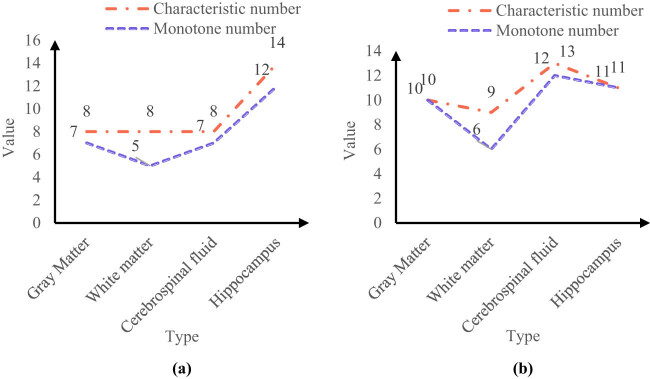
The number of features filtered based on the feature selection algorithm (normal control group and MCI group). (a) Left brain and (b) right brain.

This work also studied the distribution of selected features in different anatomical structures. The number of features filtered based on this feature selection algorithm (normal control group and MCI group) is shown in Figure 7.

It can be known from Figure 7(a) that in the normal control group and MCI group, the characteristic numbers of gray matter, white matter, cerebrospinal fluid, and hippocampus in the left brain were 8, 8, 8, and 14, and the monotone numbers were 7, 5, 7, and 12, respectively.

It can be seen from Figure 7(b) that in the normal control group and MCI group, the characteristic numbers of gray matter, white matter, cerebrospinal fluid, and hippocampus of the right brain were 10, 9, 13, and 11, and the monotone numbers were 10, 6, 12, and 11, respectively.

Table 2 is the relevant data of the number of features (normal control group and AD group) filtered based on the feature selection algorithm in this study.

**Table 2:** Number of features filtered based on this feature selection algorithm (normal control group and AD group)

Type	Left brain		Right brain	
	Characteristic number	Monotone number	Characteristic number	Monotone number
Gray Matter	9	9	8	8
White matter	7	6	10	3
Cerebrospinal fluid	9	7	9	8
Hippocampus	10	8	12	11

It can be seen from Table 2 that in the normal control group and AD group, the characteristic numbers of gray matter, white matter, cerebrospinal fluid ,and hippocampus of the left brain were 9, 7, 9, and 10, and the monotone numbers were 9, 6, 7, and 8, respectively. The characteristic numbers of gray matter, white matter, cerebrospinal fluid, and hippocampus in the right brain were 8, 10, 9, and 12, and the monotonic numbers were 8, 3, 8, and 11, respectively.

From the data, it can be seen that the texture features selected after feature selection were distributed on the four anatomies based on the normal control group and AD group. There was significant asymmetry in the selected features, that is, in the same anatomy, the distribution of the number of selected features in the left and right brain was not the same.

According to the classification of normal control group and AD group, and the classification of normal control group and MCI group, the number of selected features of each type was different in anatomy. This showed that the influence of various characteristics was different in different types of classification. In addition, among the above traits, there was no significant difference in the number of characteristics between the left and right parts. However, for the classification of normal control group and MCI, as well as the classification of normal control group and AD, the excellent characteristics of screening were different. This indicated that the asymmetry of the normal control group showed obvious asymmetry after the MCI to AD stage. It was proved from one side that this asymmetry can well describe the occurrence of the pathological process.

## 4 Conclusion

MR imaging is an important means of early diagnosis of AD, and its morphology and texture are related to it. However, the published image texture feature types are good, and the collected image features have not been effectively integrated, so the image recognition accuracy has been improved to a certain extent. On this basis, combined with the characteristics of multiple MR images, this study adopted a new image fusion method to deeply fuse different morphological and texture features in multiple different anatomical images, which significantly improved the recognition accuracy of the image. Although this study has made better improvements, there are still many problems to be discussed in depth. In addition to white matter, gray matter, cerebrospinal fluid, and hippocampus, anatomical asymmetric features of the brain, parietal lobes, and gyrus should be further explored. In order to make feature learning more intelligent and efficient, deep learning techniques can be used to improve the existing fusion algorithms.
